# Pharmacodynamics and pharmacokinetics: An unapproached issue when defining psychiatric refractory disease in the context of euthanasia

**DOI:** 10.1371/journal.pmen.0000152

**Published:** 2024-10-16

**Authors:** Luís Fonseca, Guilhermina Rêgo, Rui Nunes

**Affiliations:** 1 Bioethics Department, Faculty of Medicine, University of Porto, Porto, Portugal; 2 Psychiatry Department, Senhora da Oliveira Hospital Guimarães, Portugal; PLOS: Public Library of Science, UNITED KINGDOM OF GREAT BRITAIN AND NORTHERN IRELAND

Currently, the access of psychiatric patients to euthanasia (the act of deliberately ending patients’ lives at their explicit request to relieve unbearable suffering secondary to an incurable illness) remains controversial and far from reaching a consensus among the medical and scientific community.

The challenges are varied. The lack of ancillary tools [1] not only to make clear-cut diagnoses, but to classify illnesses’ severity, the heterogeneity of psychiatric assessments and disorders’ presentations that lead psychiatrists to assess the gravity of disease differently [2, 3], the patients’ often inconsistent capacity for well-informed decisions and consent [4] and the fact that even mild psychiatric conditions can lead to a worse quality of life [5] are some of the issues that impede a concise determination of refractory disease and unbearable suffering in mental health. Furthermore, many times, not all the necessary treatments may be available to patients [6]. In psychiatry, therapeutic approaches other than biological are extremely important [7, 8]. However, in clinical practice, the absence of clinical improvement after trials of psychopharmacology is often sufficient to consider a psychiatric disorder refractory [9]. Taking as an example Major Depressive Disorder, one of the most common psychiatric disorders worldwide [10], some guidelines have been trying to tackle the issue, but with little consistency, dubious scientific evidence and some arbitrariness [11].

One particular and critical shortcoming that is not often reported, but that we want to address here, is related to pharmacodynamics and pharmacokinetics, as there is still an incomplete knowledge of the detailed pharmacodynamics of antidepressants and a generalized unavailability of pharmacokinetic profiles of patients to guarantee targeted and tailored prescriptions. Prescriptions are still based on the psychiatrist’s experience and a trial-and-error approach [12]. This is why, for example, Escitalopram can fully remit depressive and anxiety symptoms of the same severity in one patient and not in another, even when they are of the same gender and age and have similar causal factors for their symptoms. The problem becomes more complicated with older patients where polypharmacy is common, and drug interactions can alter the plasmatic doses of medications, either augmenting the risk of side effects or averting the desirable therapeutic effect [13].

Let us imagine the following situation. A psychiatrist medicates a patient with Major Depressive Disorder using an antidepressant following their best judgment and in compliance with the best practices and guidelines available. After symptomatic remission failure, the psychiatrist decides to change the antidepressant, again following their best judgment and in compliance with the best practices and guidelines. However, the second treatment also fails. Now, a consensual definition of therapeutic resistance depression has not been established yet. However, the inadequate response to at least two antidepressant trials is generally accepted to consider the disease refractory [14]. In our practical case, assuming no psychosocial factors or comorbidities are preventing the remission, in the absence of a pharmacokinetic profile and without knowing if the drugs used reached the appropriate plasmatic dose for a therapeutic effect, how can we be sure that the chosen antidepressants were the most suitable? Should we classify the illness as refractory? If the patient advocates intolerable suffering due to refractory depression and requests euthanasia, should it be granted? Of course, these pharmacokinetic shortcomings are not exclusive to psychiatry. However, in other medical specialities, the existence of a wide range of biological markers and ancillary diagnostic tests frankly mitigate them and facilitate the assessment of whether a particular disease is refractory to treatment and causes uncontrollable pain and suffering.

The heterogeneity of psychiatric disorders and assessments and the non-existence of a clear-cut consensus on refractory disease are only a few examples of the difficulties surrounding psychiatric illnesses when euthanasia is on the table.

Therefore, especially in countries where euthanasia is available for psychiatric patients, enhancing the accuracy of the diagnosis of refractory disease and unbearable suffering, is of the utmost importance to guarantee that, in every circumstance:

- all of the necessary treatments were implemented and are universally available for every patient;
- the patients are assessed by several psychiatrists, who must, of course, have the information regarding previously used treatments but should not know whether the disorder was considered refractory by their colleagues;
- any psychiatrist must be able to request patients’ available genetic pharmacokinetic profiles and plasmatic measurements of the drugs used.

There will always be opponents and supporters of euthanasia in psychiatric patients, no matter the technological advances, scientific knowledge and diagnostic accuracy. However, it is essential to define clear-cut international and universalized criteria for refractory psychiatric disease and mental unbearable suffering. Otherwise, access inequity will contribute to worsen the already existing problems.

To grant euthanasia to psychiatric patients without these assurances is dangerous as patients with potentially curable or treatable diseases, with the opportunity of living meaningful and productive lives, could be completing suicide aided by the State.
